# Report on the New Test for Arsenic, and Its Value Compared with the Other Methods of Detecting That Poison

**Published:** 1843-07

**Authors:** Alfred S. Taylor

**Affiliations:** Lecturer on Chemistry and Medical Jurisprudence at Guy's Hospital


### REPORT ON THE NEW TEST FOR ARSENIC,

*And its Value compared with the other Methods of detecting that Poison.*

By Alfred S. Taylor,
Lecturer on Chemistry and Medical Jurisprudence at Guy's Hospital.

There is no branch of chemical toxicology which has received so much attention as that which relates to the processes for detecting arsenic in simple solutions, and in solids or liquids of an organic nature. The ingenious discovery of Mr. Marsh in 1836 threw open a new field of investigation; and by means of his apparatus, arsenic was detected in substances in which it was not even previously suspected to exist. In adopting processes for fixing the arsenic, Orfila, Danger, and Flandin, succeeded by this apparatus in detecting arsenic in cases of poisoning in the blood, liquids, and solid organs of the body. Experimentalists were, however, induced to rely upon minute stains in deposits, insusceptible of having their nature verified by other chemical processes, and this led to numerous mistakes. Thus arsenic was pronounced to be a natural constituent of the human body; it was said to exist naturally in the bones, and probably in the muscles, and in the soil of cemeteries; but more accurate analyses have since shown that these conclusions were too hastily drawn, and that we do not find arsenic in the body except in those cases where the person has died from the effects of the poison. On the other hand, it was objected to Marsh's test, that in no case could it be relied on as furnishing conclusive evidence of the presence of the poison, because the materials employed generally contained traces of that substance. Indeed it has been asserted by some that no specimen of zinc can be procured entirely free from arsenic; an assertion, however, in the truth of which, in a practical view, it is impossible to agree. Zinc may be procured so pure as to indicate, when used in Marsh's tube, not the slightest trace of the presence of arsenic; and although by using larger quantities of the metal, and employing more delicate methods of testing, some minute portion of arsenic might be found to be present in it, it is obvious that this cannot affect the practical bearing of the question. We have really to consider how far these infinitesimal traces of arsenic, admitting them to be present, may form an objection to Marsh's test, as it is commonly applied? Most toxicologists have satisfied themselves that with proper precautions all such objections may be removed: but it cannot be denied that unless the purity of the materials be carefully looked to, serious fallacies may arise; and that it would not be proper in any medico-legal case to rely simply upon a deposit on glass as evidence of arsenic being present. The real nature of the deposit requires to be confirmed by other experiments; for the physical characters are fallacious; and however the deposits from antimony may be distinguished from those of arsenic, the stains produced by selenium so closely resemble the minute stains from arsenic as to be very easily mistaken for them. It is, therefore, satisfactory that by the late discovery of Hugo Reinsch it is now in the power of the analyst readily to separate arsenic in the metallic state from organic and other liquids, and in the course of a few minutes to transform this into arsenious acid, and render it fitted for the application of sulphuretted hydrogen and other well-known tests for the poison.

Reinsch remarked that a slip of bright metallic copper which he had placed in common muriatic acid became in a short time coated with a metallic film of an iron-grey colour; and when thus coated the metal resisted for a short time the action of strong nitric acid, although it was slowly dissolved like ordinary copper. On examining the muriatic acid by sulphuretted hydrogen gas, it was found to contain arsenic in the proportion of about 0·16 per cent. of the metal. This led to the idea that copper and muriatic acid might be made a means of separating arsenic in the metallic state from liquids. When metallic copper, previously cleaned by nitric acid and polished by friction, was placed in a close vessel containing arsenical muriatic acid of s. g. 1·172, the deposit took place slowly; and it was only after the lapse of several weeks that the film of arsenic deposited on the copper presented a bright metallic lustre. When the acid was diluted with its weight of water and the mixture exposed to the air the deposit took place in a few hours. On heating the acid liquid, whether concentrated or diluted, the deposit occurred immediately. A grey pellicle was formed; but if the arsenic was in large quantity it became dark coloured at the boiling point, and then separated in black scales. It was found that this precipitation of arsenic on copper took place, although more slowly, when the arsenical compound was in extremely minute proportion; and that it would separate the metal even where a current of sulphuretted hydrogen gas failed to detect its presence.

It was next observed that when copper was boiled in a solution of arsenious acid there was no arsenical deposit formed upon it; but that if one or two drops of muriatic acid were added the copper immediately assumed the aspect of iron. According to Reinsch a quantitative analysis of arsenic might be thus made; for as the metal is separated from the copper by long boiling in the acid liquid, the quantity might be determined by the loss: to this, however, there is the objection that a quantity of chloride of copper is at the same time formed. By the use of copper, the whole of the arsenic, contained in an impure specimen of muriatic acid, may be separated from it. There are various processes by which the arsenic may be removed from the copper, as in dissolving it off by nitric acid,—heating the copper coated with arsenic in a closed tube, whereby the metal is sublimed into crystals of arsenious acid, or heating it in a tube through which a current of hydrogen is passing, in which case the arsenic is sublimed in its metallic state.

There are certain objections to this mode of testing. 1. *Antimony.* This metal affects this test as it does that of Marsh. Antimony is precipitated on copper under the same circumstances as arsenic, and here again no effect takes place until muriatic acid be added. Reinsch, however, states that antimony may be known by the metallic film having a rich violet colour, and the only case in which the arsenical deposit assumes the same tint is where the poison exists in very minute proportion. It will be seen presently that this criterion is not so well marked as the statement of Reinsch would lead us to suppose. *Tin.* Copper underwent no change in contact with a solution of this metal; mixed with a large quantity of muriatic acid until it was warmed, when there were faint traces of a metallic precipitate. Diluted solutions produced no effect whatever. *Lead.* The results obtained with this metal were much the same as those procured with tin. On boiling a salt of lead with muriatic acid there was no metallic deposit on the copper. *Bismuth.* When copper is boiled in a solution of subnitrate of bismuth, mixed with its weight of muriatic acid, a gray metallic deposit is formed on the metal, and on heating the mixture bismuth is deposited on the copper in the form of a crystalline efflorescence, whereby the bismuthic deposit may be known from that produced by arsenic and other metals. *Mercury.* On boiling copper in a weak solution of corrosive sublimate it is speedily covered with a silvery white pellicle, which by the microscope may be easily recognized as consisting of globules of mercury. *Silver.* This metal was found to give a crystalline metallic deposit on copper; although the addition of muriatic acid occasioned the separation of the greater part of the silver as chloride. From the above experiments Reinsch concluded, 1, That metallic copper was the most certain and sensitive test for arsenic, and that the reaction was very evident when the arsenic formed only $\frac{1}{200000}$ part of the solution. 2. That with regard to other metals deposited on copper under similar circumstances, these deposits might be easily distinguished from that of arsenic by their physical or other characters.

In carrying on his experiments he found that the separation of arsenic by means of copper was so complete that Marsh's test failed to show the presence of the smallest trace of arsenic in the residuary liquid. Bismuth and antimony were the only two metals deposited on copper which were likely to be mistaken for arsenic; the bismuth was always (?) deposited in a crystalline form while the antimony had a violet tint in diluted and a white or grey colour in concentrated solutions. M. Reinsch ascertained that copper would detect arsenic in a smaller proportion than he had at first supposed; in short, he found that one millionth part of a grain of arsenious acid contained in a liquid, when boiled with muriatic acid, produced a thin arsenical deposit on copper in from a quarter to half an hour. As a common experiment, a slip of bright copper was similarly treated with muriatic acid only, but it underwent no change. The surface of metallic copper is, however, apt to acquire a tarnish or dark colour, probably from the formation of some chloride of copper when left in the acid for some hours and exposed to the air. This, however, could not be mistaken for an arsenical deposit, since the latter is always completed within half an hour.

In liquids containing organic matter this separation was found to be equally complete and satisfactory. Half a grain of arsenious acid was mixed with potatoes, milk and broth, and the whole was digested with pure muriatic acid diluted with its weight of water. The liquid was boiled and filtered, and slips of copper were introduced into the filtered portion; they were immediately covered with arsenic, the deposit taking place as readily as if the poison had been dissolved in pure water. The arsenic was then converted into arsenious acid by heating the slip of copper in a tube, about thirteen inches long, drawn out to a fine point at one end. This conversion was readily effected by gently breathing into the tube at the time the heat was applied. The arsenious acid thus reproduced was dissolved in water and the usual tests applied.

Among the obvious advantages of Reinsch's test for arsenic are, 1, its extreme simplicity and the consequent facility with which it may be applied; and 2d, the rapidity with which an analysis of the most complex solid or liquid may be performed. Indeed the analysis of the contents of the stomach of a person, who is suspected to have died from arsenic, may now, as it were, form part of the post-mortem examination. Having repeated the whole of Reinsch's experiments, and applied the test in other ways, I shall here give the results.

It is not necessary to dissolve arsenious acid by long boiling in water in order to separate the arsenic in the metallic state by means of copper. If we place half a grain or a grain of the white powder in a small quantity of water, and boil in it a piece of polished copper foil, there is no effect; but if one or two drops of strong and pure muriatic acid be added the arsenic is instantly deposited, and when entirely coated, the copper should be removed, washed in water, and dried. A quantity of metallic arsenic may be thus collected, and this may be converted into distinct but small octohedral crystals of arsenious acid by heating the coated pieces of copper in a tube about four inches long and a quarter of an inch in diameter; the tube should be closed at one end and the heat gradually applied. The arsenious acid thus formed may be dissolved in water and tested with sulphuretted hydrogen gas and ammonio-nitrate of silver. The arsenites, the arseniates, arsenic acid, orpiment, and in short all arsenical compounds, were thus examined with equally satisfactory results; each experiment not occupying more than a few minutes, and the quantities used being extremely small.

Instead of copper foil, copper wire, and in some experiments the finest copper gauze was employed; but in no case were the effects so clear and decisive as with thin copper foil cut into pieces, about an inch long and the eighth of an inch in width. On boiling the copper with dilute nitric or dilute sulphuric acid, and adding a solution of arsenious acid, there was no deposit formed on it; hence the test will serve to distinguish muriatic acid from the nitric and sulphuric, by boiling the suspected acid with metallic copper and arsenious acid. The result is not always satisfactory, if the muriatic acid be in very small proportion. A chloride may be also thus distinguished from other salts; for as yet it does not appear that there is any acid but the muriatic, which will lead to the deposit of arsenic on copper.

A strong objection to the materials used in Marsh's test is, that they are apt to contain arsenic. The copper test will here assist us in determining their purity. I have tried in this way several specimens of zinc and sulphuric acid: there was no deposit on the copper, but when a minute fractional quantity of arsenious acid was added, the surface became instantly dull, and a film of metallic arsenic appeared. Again, it is possible to determine on the spot whether the matters vomited by a person from the stomach do or do not contain arsenic. A few drops boiled with muriatic acid and copper would enable the practitioner to determine the question, and to decide on the progress of the case. The same experiment might be performed with the matters evacuated. So the efficacy of the so-called iron antidote may be in this way tested. If the arsenic be not found in the supernatant liquid, when the hydrated oxide of iron has been mixed with a clear filtered solution of arsenious acid, it may be discovered by boiling a few grains of the oxide in diluted muriatic acid and adding metallic copper. According to my observation, the hydrated oxide of iron has no effect upon arsenious acid when in a state of coarse or fine powder, the state in which it is most commonly taken as a poison: it precipitates the arsenious acid in an insoluble form only, when added in very large quantity to a clear filtered aqueous solution of that substance. Reinsch's test will enable those who treat cases of poisoning to determine readily whether or not the oxide of iron be readily combined with any portion of arsenic, when expelled from the stomach.

It is right to examine the objections which may be fairly advanced against this mode of testing. In the first place, the only materials used are muriatic acid, and metallic copper; and the only one which may be supposed to present any difficulty in respect to the admixture of arsenic as impurity, is *muriatic acid.* This therefore should be taken absolutely pure, and the proper way of testing it for arsenic would be to dilute it with two parts of water, and boil a portion of copper in it before trying the suspected liquid. *Antimony.* In a diluted solution of an antimonial salt, the deposit of antimony is known from that of arsenic by its violet colour, but the arsenical deposit may have this colour also, where the quantity of poison is extremely small; and I have observed that in boiling copper with the chloride of antimony, the grey deposit was almost identical with that of arsenic. So in experimenting with the acid nitrate of *bismuth*, the deposit on copper was very similar to that of arsenic: it possessed none of the crystalline characters ascribed to it by Reinsch. The protochloride and perchloride of tin gave a dark gray deposit with a purple tint, without rendering it necessary to add muriatic acid. The common sesquichloride of iron gave also a dark gray tarnish, and with nickel there was no effect. It may be proper to state, however, that on immersing a slip of polished copper in a diluted solution of a salt of nickel, it appears to acquire instantly a bright silvery coating. This is owing to an optical effect; since the red colour of the copper neutralizes the green colour of the liquid, and causes the reflection of white light; hence, on removing it the copper is seen to possess its usual red lustre. Cadmium produced no effect; copper was boiled in a solution of the nitrate to which muriatic acid had been added, and also in a solution of chloride made by dissolving the pure oxide in muriatic acid. In neither case, although the metal was left in the liquid for upwards of half an hour, was there any change. Here then we have an important difference between cadmium and arsenic: these metals resembling each other strikingly in the fact, that they yield metallic sublimates when their oxides are heated with carbonaceous matter, and yellow sulphurets, when their compounds are treated with a current of sulphuretted hydrogen gas. In respect to silver and mercury, the metallic deposits are formed without the addition of muriatic acid; although in a very diluted solution of a mercurial salt, the deposit would take place very slowly, unless muriatic acid were added.

The appearance of the deposit in each case, is very different to that formed by arsenic. Lastly, the alkaline sulphurets, or any liquid containing sulphuretted hydrogen or sulphur evolved in decomposition, will give a tarnish to copper, varying in tint, which might be easily mistaken for an arsenical deposit where the quantity of arsenious acid present, was small. Indeed, in several experiments specially made, it was impossible by reference to physical characters only, to distinguish the stain produced by the sulphuret from that formed by arsenic. There is, however, this marked difference that the tarnish from sulphur takes place sooner or later, on mere contact with the liquid, or on warming it without the addition of muriatic acid; while in the case of arsenic, the addition of this acid is absolutely necessary or no deposit will take place. There are besides other and ready means of discovering the presence of the smallest traces of a sulphuret as of sulphuretted hydrogen gas in a liquid.

The only real objections to this test, therefore, refer to the possible deposit on coppers of other metals or substances which might be mistaken for arsenic. The above facts will, it appears to me, justify the conclusion that, from physical characters alone, it would be hazardous to pronounce on the presence of arsenic in a suspected unknown liquid. But here we come to the extreme value of this most useful test; the arsenic may be easily obtained from the copper by heating the metal in a reduction tube. We may procure it under the form of crystalline octohedra or of a white sublimate, easily dissolved by water in sufficient quantity for the application of the silver and sulphuretted hydrogen tests. The obtaining of octohedral crystals is highly characteristic of arsenious acid; since there is no other volatile poison which crystallizes in a similar form; and the octohedra of arsenic, from their great brilliancy, are visible either to the naked eye, or under a lens of moderate power, when existing in imponderable quantities.

The simple answer to all objections founded on the deposit of other metals or substances on copper is, that it is impossible to obtain by heating the metal, octohedral crystals possessing the well-known characters of arsenious acid. The limit to the practical value and application of the test then, is that point where, with a metallic deposit on copper, we are unable to procure by heat a body possessing the properties of arsenious acid. Beyond this, there may be chemical probability of the presence of arsenic from the appearance of the deposit; but it appears to me doubtful whether we should be justified in saying that we had medico-legal certainty. This admission, however, must not lead us to suppose that the test is very limited in its application; on the contrary, it will detect and separate a smaller quantity of arsenic than we shall probably ever have to encounter in a medico-legal analysis; and it is far more easy to obtain arsenious acid from a minute quantity of the metal than to obtain the metal from the reduction of a minute quantity of the sesqui-sulphuret, according to the process commonly adopted. Further, there is as much certainty with this as with Marsh's test, or other modes of testing for arsenic; and where it fails, it is pretty certain that no other test will succeed.

The most valuable application of the copper test will, however, be seen in the detection of the presence of arsenic in the most complex organic mixtures containing that poison. Medical jurists have long abandoned the use of the liquid tests in these cases; and they have relied either on Marsh's apparatus, or on the precipitation of the arsenic in the state of impure sesqui-sulphuret, and the subsequent reduction of that compound to the metallic state by flux. In the use of Marsh's apparatus, much inconvenience has been experienced from the froth; and when the quantity of arsenic was small, its diffusion through a large quantity of liquid rendered its separation troublesome. The processes of carbonization suggested by Orfila and Danger, and Flandin, removed this difficulty to a certain extent; but these processes required time and care to prevent the loss of arsenic. There could be no doubt, however, as it has been elsewhere stated, that Marsh's process was a considerable improvement upon the old plans adopted for the separation of arsenic. With respect to the precipitation of the poison by sulphuretted hydrogen, there were many difficulties which the experimentalist had to encounter. The precipitation was slow, the sulphuret was commonly mixed with much organic matter, so that on attempting to obtain from it the metal, the sublimate was often obscured by the foreign substances with which it was mixed. If the proportion of sesqui-sulphuret were small, it could not be wholly separated from the liquid; or if it were thus separated, the quantity was not sufficient to yield a distinct and well-defined sublimate of metallic arsenic. In short, those who have tried both processes must have perceived that there were many inconveniences connected with them. The following experiments will show that the copper test supplies all that is required to render the search after arsenic simple, speedy, and certain.

The $\frac{1}{144}$th part of a grain of arsenious acid was mixed with two fluid drachms of milk. The mixture was boiled with a few drops of muriatic acid, and a slip of copper was introduced. In less than a minute the metal was coated with a grey film of metallic arsenic. Several pieces were thus coated; they were washed in water, dried in the heated current of air over a spirit lamp-flame, and introduced into a small reduction tube. On applying a gentle heat to the copper, octohedral crystals were obtained; visible to the eye in the light of the sun, but plainly distinguishable with a lens of low power. The crystals dissolved in water gave the usual reactions with the ammonio-nitrate of silver and sulphuretted hydrogen gas. In a second experiment, the same quantity of arsenic was mixed with two drachms of porter; in a third, a like quantity with two drachms of gruel, and the copper test was ap plied with similar satisfactory results. Each experiment was completed in about five minutes. Brandy, containing a poisonous impregnation of arsenic, was then tested; and the arsenic readily separated. A few drops taken from a bottle of port wine, containing arsenic, and which had nearly caused the death of three persons about four years ago, were next submitted to the test, and the metal readily obtained. In organic solids the results were equally striking: a few grains of a cake containing arsenic, which had been used in an attempt to poison, were boiled in a small quantity of distilled water; and muriatic acid and copper added, when metallic arsenic was immediately precipitated. The contents of the stomach of a person who was poisoned by arsenic in February, 1834, which had been loosely exposed, and allowed to become decomposed during a period of upwards of nine years, were next examined. A few drops of the thick turbid liquid were placed in a tube and boiled with dilute muriatic acid and water; the copper introduced was covered with a bright layer of metallic arsenic. The contents of three other stomachs taken from persons who were poisoned by arsenic in 1835, 1838, and 1840, gave precisely similar results. In the last case, the whole of the contents with the food at the time contained in the organ, had been evaporated to a dry solid mass; a few grains of this were sufficient to furnish a clear demonstration of the presence of arsenic by the aid of the test. In the conversion of the metal to arsenious acid, it will at once suggest itself that if octohedral crystals should not be obtained by heating one portion of copper, several slips should be introduced together or successively. In all these cases, arsenic would have been discovered by the application of Marsh's test, or sulphuretted hydrogen gas; but the process would have occupied a much longer time, and with regard to Marsh's test the metallic arsenic could not have been so speedily converted to arsenious acid in a form convenient for the identification of its properties.

There is no doubt that this is an extremely delicate test; but there is a want of precision about the statements of M. Reinsch on this subject. He asserts that he has been able to detect by means of it $\frac{1}{1000000}$th of a grain of arsenious acid. It appears, however, probable that by this we are to understand he separated the arsenic by copper, where it formed only one millionth part of the solution by weight. In order to obtain the millionth of a grain of arsenic, it would be necessary to take *one drop* of a solution formed by dissolving one grain of arsenic in sixteen gallons of water, or their equivalent fractional parts. In these estimates it should always be clearly stated, not only what the absolute weight of the poison may be, but the actual quantity of water in which it is diffused; for chemical tests cease to act from two causes; first, from the very minute quantity of poison present; and secondly, from the large quantity of menstruum in which it may be diffused. In proof of this it may be stated, that if the same weight of arsenic be dissolved in two different quantities of liquid, a test which will clearly indicate its presence in the one case, will fail to do so in the other. Thus in one experiment the $\frac{1}{400}$th grain of arsenious acid, dissolved in twenty drops, or 8000 times its weight of water, was immediately affected by a current of sulphuretted hydrogen gas; but upon applying the test to $\frac{1}{400}$th grain dissolved in four drachms or 100,000 times its weight of water, the liquid scarcely acquired any perceptible tinge; and had the dilution been carried to a million times, there would have been probably no visible change whatever. The same rule applies to other tests, and when, therefore, we speak of a test detecting or separating the millionth part of a grain of arsenic, the whole of the circumstances should be explicitly stated. The delicacy of Marsh's test has often been estimated by the probable or assumed weight of the sublimate obtained; but there appears to be an error in this mode of estimating the relative efficacy of tests; because it is quite certain that a deposit of arsenic may be procured by Marsh's apparatus, which, if it were transferred to the tube, would give no indication whatever of its presence from its extremely minute quantity. The truth is, in this case we are operating upon the whole quantity of poison, dividing and subdividing the metal into a series of infinitesimal deposits, the weight of some of which might not be equal to the millionth part of the weight of the poison which is furnishing them.

The results which I obtained with respect to the tests for arsenic are that the sulphuretted hydrogen gas begins to produce a reaction indicative of arsenic, when the weight of arsenious acid is not more than $\frac{1}{4000}$th part of a grain in ten drops of water; the arsenic thus forming $\frac{1}{40000}$th part of the liquid. With the $\frac{1}{2000}$th grain, and a smaller quantity of water, the effect was much more decided. With respect to Marsh's test, as the poison was mixed with the whole of the acidulated liquid, the dilution was disproportionately great. No well defined deposits were procured with $\frac{1}{2000}$th of a grain in two ounces of water; but faint brown rings began to appear where, with the same quantity of water, the proportion of arsenious acid reached $\frac{1}{1000}$th of a grain. In the use of Reinsch's test, we are not obliged to dilute the liquid with more water than may be just sufficient to cover the copper; and there is no loss in these minute quantities, a circumstance which necessarily happens with Marsh's test, during the combustion of the arsenuretted hydrogen. The copper test failed to detect $\frac{1}{400}$th grain in thirty drops of water, the dilution being equal to 120,000 times the weight of the arsenic. The deposit commenced with a faint violet coloured film, when the quantity of arsenious acid was equal to $\frac{1}{3000}$th grain in thirty drops of water, or under a dilution of 90,000 times its weight; it was also very decided with the $\frac{1}{2000}$th of a grain in the same quantity of water, but in neither of these cases could octohedral crystals of arsenious acid be obtained. The following experiment will show how much this test is influenced by dilution: the copper was coated in a few minutes when boiled in a solution containing $\frac{1}{4000}$th grain of arsenious acid in ten drops of water, although the test had completely failed to detect the same weight of arsenic, in three times that quantity of water. There is no doubt that by preventing the dilution of the poisonous liquid, Marsh's apparatus would detect a much smaller quantity of arsenic than has here been stated. This has been contrived by Mr. Marsh, in inclosing the liquid for analysis in a small piece of glass-tube, distinct from the general liquid of the apparatus, which acts by confining the gas.

The question really is, however, At what point do the tests fail to detect the poison with certainty? And the reply appears to me to be when the results obtained by these modes of testing cannot be confirmed by other experiments applied to them. To prove that the substance deposited is arsenic, we ought to be able to procure from it arsenious acid recognizable by some at least of its usual properties. This is very easily obtained from small quantities, by the copper test. I have found that crystals of arsenious acid might be procured from so small a quantity as from the $\frac{1}{100}$th to the $\frac{1}{150}$th of a grain of arsenious acid. Whether a smaller quantity than this will yield sufficient metal for this confirmatory proof must be settled by further experiments; but it appears to me nothing short of this, whether in reference to Marsh's or Reinsch's test, will afford what the medical jurist requires, judical certainty.

Another useful application of the copper test may be made in the following way. If the arsenic have been thrown down from an organic liquid in the form of impure sesquisulphuret, this may be dried and deflagrated with nitre, or decomposed by nitromuriatic acid, whereby arseniate of potash or arsenic acid will result, and the organic matter will be entirely decomposed. In the case of deflagration by nitre, the surplus nitric acid should be expelled by sulphuric acid, and the arseniate dissolved out of the residue; or if nitro-muriatic acid be used, the liquid may be evaporated to dryness. On boiling either of these products with copper and muriatic acid, the metallic arsenic will be readily procured.

This test will no doubt be fully examined by others. Without saying that it will at once supersede all other modes of testing for arsenic, it appears to me to surpass them in certainty and simplicity, and in its ready application to the detection of the poison in all forms of mixture and combination. In its principle it is rather the reverse of the method which has been hitherto pursued; for by it we procure the metal first, and produce the compound to be afterwards tested by liquid reagents. With this discovery we have now analogous processes for the detection of three important metallic poisons and their salts, namely, arsenic, mercury, and copper. The chief matter of surprise is, that a method so simple should not have been earlier discovered in relation to a poison which has received so much attention from toxicologists; more especially, as it has been long known that arsenious acid was soluble in muriatic acid.

